# *ATP1A3* Mutation in Adult Rapid-Onset Ataxia

**DOI:** 10.1371/journal.pone.0151429

**Published:** 2016-03-18

**Authors:** Kathleen J. Sweadner, Camilo Toro, Christopher T. Whitlow, Beverly M. Snively, Jared F. Cook, Laurie J. Ozelius, Thomas C. Markello, Allison Brashear

**Affiliations:** 1 Departments of Neurosurgery, Massachusetts General Hospital and Harvard Medical School, Boston, Massachusetts, United States of America; 2 NIH Undiagnosed Diseases Program, Common Fund, Office of the Director, NIH, and Office of the Clinical Director, NHGRI, Bethesda, Maryland, United States of America; 3 Departments of Radiology and Biomedical Engineering, Wake Forest School of Medicine, Winston-Salem, North Carolina, United States of America; 4 Department of Biostatistical Sciences, Wake Forest School of Medicine, Winston-Salem, North Carolina, United States of America; 5 Department of Neurology, Wake Forest School of Medicine, Winston-Salem, North Carolina, United States of America; 6 Department of Neurology, Massachusetts General Hospital, Boston Massachusetts, United States of America; 7 NIH Undiagnosed Diseases Program, Common Fund, Office of the Director, NIH, and Human Biochemical Genetics Section, Medical Genetics Branch, NHGRI, Bethesda, Maryland, United States of America; University of Tennessee Health Science Center, UNITED STATES

## Abstract

A 21-year old male presented with ataxia and dysarthria that had appeared over a period of months. Exome sequencing identified a *de novo* missense variant in *ATP1A3*, the gene encoding the α3 subunit of Na,K-ATPase. Several lines of evidence suggest that the variant is causative. *ATP1A3* mutations can cause rapid-onset dystonia-parkinsonism (RDP) with a similar age and speed of onset, as well as severe diseases of infancy. The patient’s *ATP1A3* p.Gly316Ser mutation was validated in the laboratory by the impaired ability of the expressed protein to support the growth of cultured cells. In a crystal structure of Na,K-ATPase, the mutated amino acid was directly apposed to a different amino acid mutated in RDP. Clinical evaluation showed that the patient had many characteristics of RDP, however he had minimal fixed dystonia, a defining symptom of RDP. Successive magnetic resonance imaging (MRI) revealed progressive cerebellar atrophy, explaining the ataxia. The absence of dystonia in the presence of other RDP symptoms corroborates other evidence that the cerebellum contributes importantly to dystonia pathophysiology. We discuss the possibility that a second *de novo* variant, in ubiquilin 4 (*UBQLN4*), a ubiquitin pathway component, contributed to the cerebellar neurodegenerative phenotype and differentiated the disease from other manifestations of *ATP1A3* mutations. We also show that a homozygous variant in *GPRIN1* (G protein-regulated inducer of neurite outgrowth 1) deletes a motif with multiple copies and is unlikely to be causative.

## Introduction

A patient with unexpected adult onset of ataxia and rapid deterioration had exome sequencing performed by the NIH Undiagnosed Diseases Program [1], which uncovered candidate gene mutations. A *de novo* mutation was found in *ATP1A3*, which encodes the α3 subunit isoform of the Na,K-ATPase catalytic subunit. Of the three isoforms of the catalytic (α) subunit expressed in the CNS, α3 is found only in neurons [2]. Na,K-ATPase transports Na^+^ out of the cell and K^+^ into the cell. This isoform is so important to brain function that dominantly-inherited mutations can produce cognitive impairment, developmental delay, psychiatric disorders, seizures, and several specific syndromes that differ in severity, age of onset, and triggers [3–7]. Mutations in *ATP1A3* produce rapid-onset dystonia-parkinsonism (RDP) [8]; alternating hemiplegia of childhood (AHC) [9], and severe infantile epilepsy [10,11]. Ataxia has appeared with *ATP1A3* mutations in a syndrome with cerebellar ataxia, areflexia, pes cavus, optic nerve atrophy, and sensorimotor deafness (CAPOS) [12], and in pediatric cases where febrile episodes resulted in relapsing ataxia combined variably with symptoms shared with RDP and/or AHC [13–16], including one patient with an ataxia episode as an adult [15]. In RDP, disease onset often follows a stressful trigger and usually develops over a period of hours to weeks or months. Neuropathology has been described in aged RDP patients [17], however none of the *ATP1A3*-related diseases have been considered neurodegenerative. We expressed the patient’s novel *de novo ATP1A3* mutation and tested its function to investigate its potential pathogenicity.

A second *de novo* variant was in *UBQLN4*, which encodes ubiquilin 4, an adaptor protein involved in ubiquitin-directed protein quality control. The *UBQLN4* homologs *UBQLN1* and *UBQLN2* have been implicated in the pathogenesis of neurodegenerative diseases, i.e. Parkinson’s, Alzheimer’s, Huntington's, and amyotrophic lateral sclerosis/frontotemporal dementia [18]. The patient’s *UBQLN4* variant was compared to previously-identified causative mutations in its homologs, and similarity supports the idea that it may have contributed to the atypical ataxia of this individual as part of a response to misfolded protein. An inherited deletion in *GPRIN1*, however, showed genetic and sequence characteristics of a tolerated variant.

## Subjects and Methods

### Clinical evaluation

The patient had clinical evaluations initially at NIH and later at Wake Forest School of Medicine, where a battery of tests developed for RDP patients ([14,15] and S1 File) was administered. At the NIH, the patient and his nuclear family were evaluated under the auspices of the NIH Undiagnosed Diseases Program (UDP) and all were enrolled under NIH protocol 76-HG-0238, “Diagnosis and Treatment of Patients with Inborn Errors of Metabolism and Other Genetic Disorders”, a study approved and monitored by the Intramural NHGRI Ethics Review Board. At Wake Forest, the study was approved by the Wake Forest School of Medicine Institutional Review Board. All participants were adult and capable of informed consent, and written informed consent was obtained.

### Genetic studies

High-density SNP mapping and exome sequencing were performed on the patient, his parents (of Swiss origin), and sibling. Variants were filtered by a multi-step pipeline developed by the Undiagnosed Diseases Program that utilizes haplotype mapping and pedigree structure, and filters variants by multiple technical criteria and different inheritance models [19]. Variants of interest were validated by Sanger sequencing.

### Mutation structure and function

Na,K-ATPase α1 and α3 are 88% identical. Homologous residues were identified in the crystal structures of *ATP1A1* Na,K-ATPase in the K^+^-bound and Na^+^-bound conformations (PDB ID’s 2ZXE and 3WGU) [20,21] and analyzed with the Swiss PDV Viewer, SPDBV 4.1. The p.Gly316Ser mutation was introduced into human *ATP1A3* cDNA in an expression vector previously mutated to be resistant to the inhibitor ouabain [8]. It was expressed transiently in HEK293 cells, which have endogenous ouabain-sensitive *ATP1A1*. An established cell survival assay was employed to determine whether the introduced *ATP1A3* Na,K-ATPase is active enough to functionally replace the endogenous *ATP1A1* Na,K-ATPase when it is inhibited [8]. Gel electrophoresis and staining with ATP1A3-specific antibodies were performed as described [10].

## Results

### Phenotype

The patient had normal gestation, delivery and early development except for mild amblyopia. His gross and fine motor developmental milestones were achieved on target, but speech was delayed until 2 years of age. He exhibited a mild learning disability and dyslexia, but no developmental regression. At age 19 he had unexplained episodes of vertigo lasting days, which resolved. Difficulties with balance and gait, slurred speech and drooling emerged at age 21 and worsened during a summer spent away from home. A tremor of the hands (primarily action tremor) was subsequently noticed as well. These symptoms progressed over the next 6 months with profound dysarthria and ataxia that led to the use of a wheelchair. The patient’s mother reported that the symptoms progressed most rapidly over the last 2 months of this period and then stabilized.

**Fig 1:**
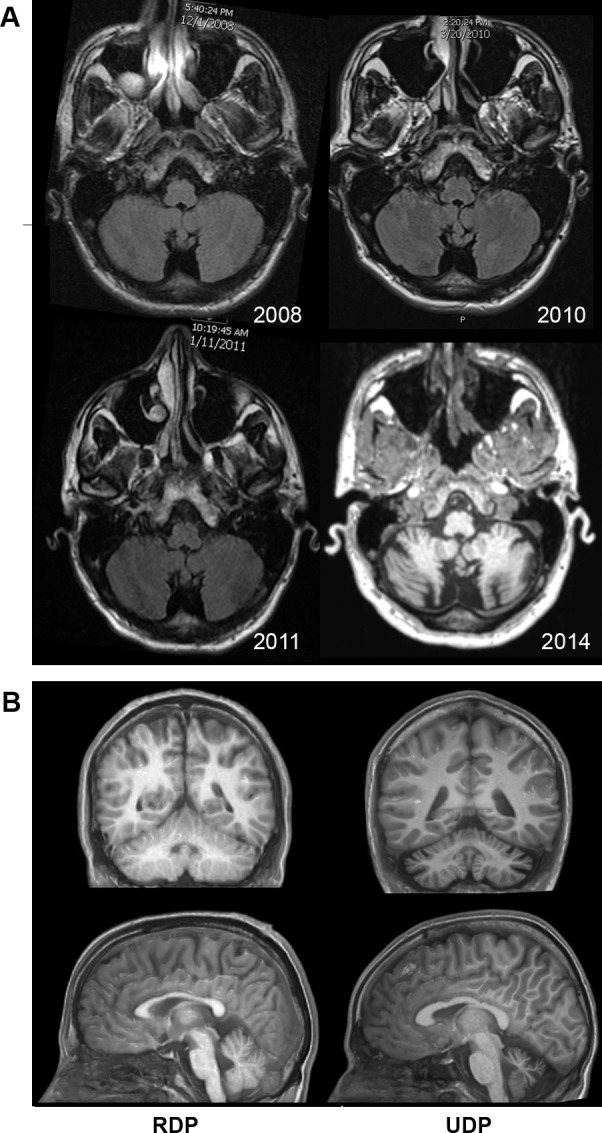
Sequential and comparative MRI. (A) Axial T1-weighted images from successive MRI scans acquired from 2008 (year of onset) to 2014 demonstrate the progressive development of cerebellar atrophy. (B) Coronal (top row) and sagittal (bottom row) T1-weighted images from MRI demonstrate relatively normal cerebellar structure and volume of a representative RDP patient (left images) in contrast to marked cerebellar atrophy of the patient in 2014 (right images).

When evaluated at the NIH at age 24, the patient had findings of a moderate to severe cerebellar syndrome with limb and gait ataxia and cerebellar dysarthria. His movements were slow, speech was soft, and he ambulated with small steps. He had mildly elevated tone worse in the lower extremities. Longitudinal MRI imaging demonstrated progressive cerebellar degeneration (Fig 1A). No peripheral nervous system involvement was found by exam or electrodiagnostic studies; spinal fluid neurotransmitters and muscle biopsies were normal; and there was no benefit from a trial of levodopa: 25 mg carbidopa/100 mg levodopa 3 times a day for ~ 2 months in 2011. Testing for the following disorders were negative: encephalitis, immune disease, metabolic or mitochondrial disease, lysosomal storage diseases, and spinocerebellar ataxias.

The patient has taken a vitamin compound for the past 3 years, which the family reported benefitted speech, gait, balance, and tremor. The total daily dose is B1, 100 mg; B2, 200 mg; B5, 200 mg; C, 500 mg; E, 400 IU; folinic acid 10 mg; selenium 50 μg; coenzyme Q-10 500 mg; lipoic acid 500 mg; and biotin 10 mg.

Before identification of the gene, RDP had not been considered in the diagnosis because of the patient’s profound ataxia. *ATP1A3* mutation and rapid development of severe symptoms made an association more likely. Follow-up evaluation of the patient at age 26 at Wake Forest School of Medicine revealed partial overlap of clinical features with RDP. Video is in the S1 Video. A patient history questionnaire and neurological exam that were developed for RDP patients was administered. No substance use or chemical exposure was reported that might have triggered or caused the symptoms. The principal similarities to RDP were dysarthria, including difficulty with speech, swallowing, and drooling (common manifestations in RDP) [3], prominent bradykinesia and masked face, a history of childhood learning difficulties, and relatively rapid onset of symptoms as a young adult. The most notable differences from RDP were that ataxia predominated instead of dystonia, and there was tremor of the hands. Neuropathology and MRI of RDP patients shows detectable pathology in dentate nucleus and superior cerebellar peduncle, but not extensive atrophy [17]. In contrast, the patient at age 26 showed severe cerebellar atrophy, contrasting with an image from an age- and sex-matched RDP patient (Fig 1B). Motor test scores are listed in Table 1 compared to the mean scores of RDP patients in our database. A high score for RDP severity was based on findings of definite affected arm and bulbar muscles and that the patient uses a wheelchair.

**Table 1:** Neurological evaluation.

Test	Score	RDP average
**UPDRS-III, motor subscore**	30	31.9
**BFMS dystonia rating**	3	55.7
**IADL activities of daily living**	15	22.0
**RDP neurologic examination:**		
**dystonia**	2	3.97
**parkinsonism**	3	3.72
**RDP severity**	4	3.22

The tests administered were:

Unified Parkinson’s Disease Rating Scale (UPDRS)[22]; Burke-Fahn-Marsden Dystonia Rating Scale (BFMS)[23]; Instrumental Activities of Daily Living (IADL)[24]; RDP Neurologic Examination: dystonia and parkinsonism scores = 1, no dystonia/parkinsonism, 2, possible, 3, probable, and 4, definite. RDP severity = 1, limb dystonia only including writer’s cramp, 2, affected arm and bulbar muscles with normal gait, 3, same as 2 with legs affected but walking unassisted, and 4, same as 2 with legs affected but walking with walker or in a wheelchair.

Previously, findings of cognitive impairment and psychosis in RDP led to development of a comprehensive battery of cognitive and psychiatric tests suitable for people with motor limitations [25,26], which were administered to this patient. In the psychiatric battery (CIDI DSM-IV and ICD-10; HAM-A and–D; Y-BOCS) the patient was within normal limits, while taking clonazepam 0.5 mg daily for anxiety. A comprehensive battery of neuropsychological tests showed overall skills at the low end of average, verbal and nonverbal skills evenly matched, and memory within normal limits (verbal > nonverbal). However there was poor discrimination on delayed recognition of abstract designs, and impaired psychomotor processing speed, with performance limited by motor impairment. The data for psychiatric and cognitive testing are Tables A and B in S1 File.

### Genetics

Analysis of exome sequences of the patient and his family identified four candidate variants (Table 2). Two candidates, *ATP1A3* and *UBQLN4*, were heterozygous and de novo, and two others were inherited. The potential of *UBQLN4* and *GPRIN1* as candidates is discussed below. X-linked *SRPK3* is a member of a family of kinases that regulate RNA processing. However, in humans, mice, and pigs it is implicated in myogenesis and highly expressed in muscle but not brain [27]. Other variants that did not score as possibly causative included four additional homozygous variants inherited from the parents (*RFX5*, *FAM194*, *OR1L6*, *COMMD10*)*;* two additional hemizygous X-linked variants (*SSX3*, *USP11*); four compound heterozygous variants (*CYP2C18*, *KL*, *TTN*, *PLEC*)*;* and one inherited heterozygous variants (*KARS*).

**Table 2:** Candidate variants detected by exome sequencing.

	Chr	RefSeq	variant	patient	mother	father	brother	ref
***ATP1A3***	19	NM_152296.3	[c.946G>A, p.Gly316Ser]	A/G	G/G	G/G	G/G	G/G
***UBQLN4***	1	NM_020131.3	[c.1444G>A, p.Glu482Lys]	A/G	G/G	G/G	G/G	G/G
***GPRIN1***	5	NM_052899.2	[c.690_714delinsA, p.Glu233_Lys240insA] + [c.690_714delinsA, p.Glu233_Lys240insA]	homo-del	het-del	het-del	homo-ref	
***SRPK3***	X	NM_014370.2	[c.1373C>A, p.Thr458Asn]	hemiA	A/C	hemi C	hemi C	C/C
**status**				affected	unaffected	unaffected	unaffected	

Ref is the reference sequence, del is deletion. *ATP1A3* = α3 catalytic subunit of Na,K-ATPase; *UBQLN4* = ubiquilin-4. *GPRIN1* = G-protein-regulated inducer of neurite outgrowth 1; SRPK3 = serine arginine domain selective protein kinase 3. All variants were verified by Sanger sequencing.

**Fig 2:**
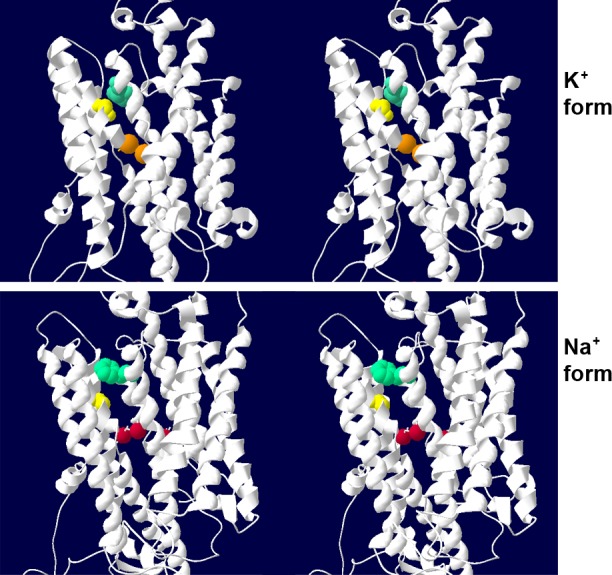
Stereo images of Gly316 and Phe780 in the K^+^ and Na^+^ conformations. The structures can be viewed in stereo by focusing the eyes behind the plane of the top or bottom image pair and letting the eyes drift separately until a central image fuses. On the top, the transmembrane domain of Na,K-ATPase is shown from a crystal structure in the E2 (K^+^-bound) conformation. Gold spheres are K^+^ ions in the ion binding pocket. The yellow space-fill residue is Gly316. Opposite it, the blue-green residue is Phe780, mutated to leucine in RDP. On the bottom, red spheres are Na^+^ ions in the ion binding pocket of a crystal structure in the E1 (Na^+^ bound) conformation. The rearrangement of transmembrane helices has separated Gly316 and Phe780 significantly.

Mutations in *ATP1A3* produce neurologic disease [28]. The protein has 10 transmembrane spans, and cycles through conformation changes that alternately bind Na^+^ or K^+^ in binding pockets in the middle of the membrane. p.Gly316Ser mutation is novel, and is in the highly conserved 4th transmembrane span. In a Na,K-ATPase crystal structure in the K^+^-bound conformation, p.Gly316Ser is directly apposed to a residue in the 5^th^ transmembrane span that is mutated in RDP (p.Phe780Leu) (Fig 2, top) [8]. The glycine is <3.7Å from the side chain of the phenylalanine, whereas the side chain of serine is polar and would clash with the phenylalanine at <2.2Å. The mutations are close to the ion binding pocket. In the Na^+^-bound conformation, in contrast, the glycine and phenylalanine are 11 Å apart because of a sliding rearrangement of the transmembrane spans (Fig 2, bottom). Contact of the mutated glycine and phenylalanine residues is thus involved in conformation change during ion pump activity, and is a credible basis for impairment of activity.

### ATP1A3 mutation validation and phenotype

**Fig 3:**
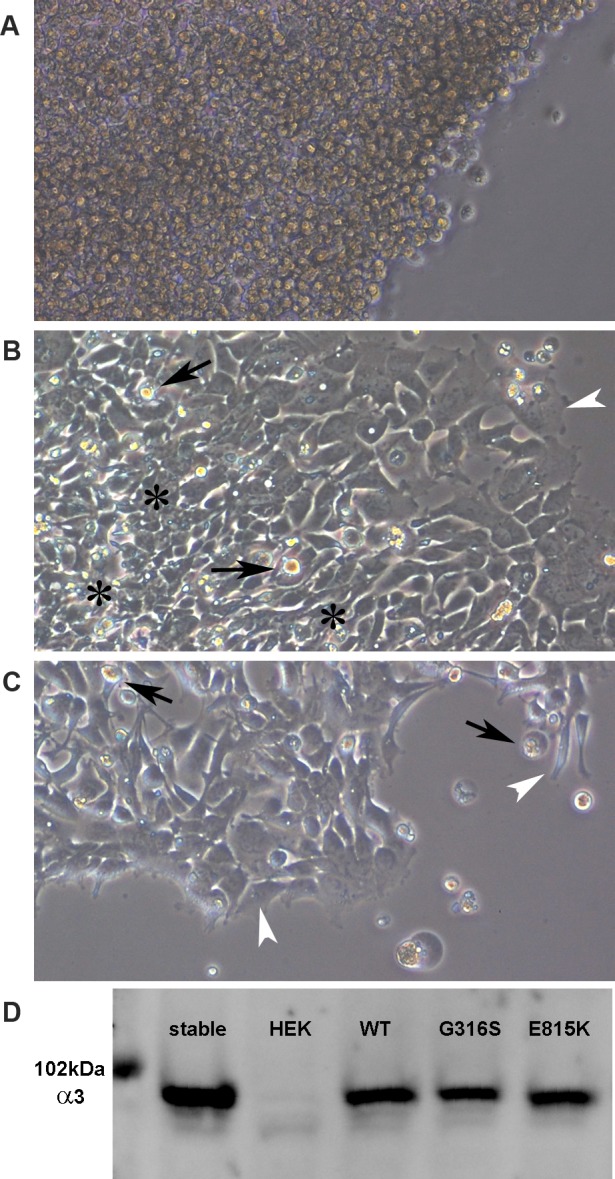
Impairment of Na,K-ATPase function. (A-C) Ouabain is a specific Na,K-ATPase inhibitor. HEK293 cells are shown 5 d after they were transfected and 4 d after 0.5 μM ouabain was added. In A, mock-transfected HEK cells had all died. In B, transfection was with unmutated *ATP1A3*. Cells survived and divided, becoming crowded (asterisks), medium acidified overnight, and some death due to overcrowding was apparent (arrows). Cells at the edge of open spaces were flattened (white arrowheads). In C, the p.Gly316Ser mutation was transfected. Many cells survived, but were unable to divide, and the medium did not acidify. Over the subsequent two weeks there was a slow attrition of cells and no detectable division of the remaining living p.Gly316Ser cells. (D) Na,K-ATPase α3 western blot of equal amounts of total protein from cell homogenates demonstrating that the mutant protein is expressed at levels comparable to controls. A stable WT *ATP1A3*-transfected cell line was a positive control for optimal *ATP1A3* expression. The visible molecular weight marker (Amersham Rainbow) was 102 kDa, and α3 migrates at ~93 kDa (faster than its molecular mass). HEK cells express only *ATP1A1*, which is not detected by the specific antibody (Santa Cruz Biotechnology sc-16052). The other lanes show transient expression of WT *ATP1A3*, p.Gly316Ser, and p.Glu815Lys, an inactive mutation found in AHC [28]. The expression results and the failure to support cell growth are representative of three experiments.

Cellular expression studies were performed to test whether the p.Gly316Ser *ATP1A3* mutation impairs the activity of the enzyme. The test is a survival assay, in which only active enzyme can keep the cells viable when their endogenous Na,K-ATPase is inhibited. The mutation was introduced into a ouabain-resistant cDNA of human *ATP1A3* and expressed in a ouabain-sensitive human cell line, HEK 293. Addition of ouabain killed control cells that expressed no *ATP1A3* (Fig 3A). Expression of wild-type, ouabain-resistant *ATP1A3* allowed cells to survive and divide normally (Fig 3B). The cultures double every 24 hours, and evidence of cell crowding (asterisks) and dying cells (black arrows) was seen. Within a few more days, cultures with WT *ATP1A3* (like untransfected cultures) were too crowded to be viable (not shown). Cells expressing the p.Gly316Ser mutation survived for 2–3 weeks in stasis but could not divide, remained uncrowded, and were gradually lost (Fig 3C). At the edges of open spaces on the plate, cells with WT *ATP1A3* were well-flattened (white arrowheads), while those with pGly316Ser were more rounded. Other p.Gly316Ser-transfected wells were trypsinized at 96 h and replated in 0.5 μM ouabain, and these had no surviving cells after 3 d (not shown). Na,K-ATPase α3 expression levels assessed by Western blot were close to those of the WT controls (Fig 3D). The data indicate that there is impaired pump activity, with just enough residual activity to maintain cell stasis, but not viability. Partial inhibition of activity, often with reduced Na^+^ affinity, has been reported for RDP mutations [28]. In contrast, mutations producing severe disease in infants (AHC or severe epilepsy) did not support cell survival at all in the same assay [10]

## Discussion

### Mutation in *ATP1A3*

This is a new phenotype associated with a novel *ATP1A3* mutation. The overlap with RDP in rapid onset and dysarthria and bradykinesia symptoms, the loss of Na,K-ATPase activity, and the conformationally-sensitive proximity of the mutated amino acid to a known RDP mutation (Fig 2) support a shared etiology. Here ataxia was adult rapid-onset, irreversible, and the predominant symptom. Ataxia frequently manifests in older children with AHC along with a variety of other symptoms [7,9,29], and it appears with other symptoms in children that first manifest symptoms after the 18 month infantile diagnostic cutoff for AHC [13]. A recent case with *ATP1A3* mutation presented with a remitting-relapsing course of acute ataxia triggered by fever [15]. Patients with CAPOS syndrome also exhibit ataxia, and it appears acutely during an illness with fever, and also has a remitting-relapsing course [12,30]. Importantly, hemispheric and vermian cerebellar atrophy is severe in our patient, but in CAPOS and most other *ATP1A3* cases, cranial MRIs are usually normal [30,31], with rare reports of vermian atrophy [15,32].

Although the neurodegenerative course here is novel, this is the third clinical case linking mutation of *ATP1A3* to neuron death. The neuropathological study of four brains from aged RDP siblings with the p.Ile758Ser mutation demonstrated neuron losses in dentate nucleus, globus pallidus, and other regions implicated in dystonia, with modest losses in cerebellar cortex [17]. In an infant with catastrophic epilepsy and p.Gly358Val *ATP1A3* mutation that severely inhibited the ATPase, cerebellar atrophy was among the neuropathological findings [10]. Whether cell death is causative of symptom onset in *ATP1A3* diseases or an occasional consequence of sustained neurophysiological abnormality is yet to be determined. Here cerebellar atrophy paralleled clinical progression.

In *ATP1A3* mutations that have been investigated for function, in vitro kinetics and oocyte studies have found only loss of function or reductions in kinetic parameters [28]. A gain of function such as development of a leak current [33] is possible but has not yet been demonstrated for *ATP1A3*, although somatic mutations in *ATP1A1* found in aldosterone-secreting adenomas induced significant leak currents [34]. Three mutations that cause severe AHC have been found to have loss of function and dominant negative effects when coexpressed with normal *ATP1A3* in oocytes [35]. Based on the critical role of the Na,K-ATPase, our patient’s p.Gly316Ser *ATP1A3* mutation may be sufficient to cause his disorder.

### A potential role for *UBQLN4*

*UBQLN4* encodes an adaptor protein linking ubiquitinated proteins and the proteasome. It is not yet associated with human disease, however the *de novo* variant of ubiquilin-4 might have a role in the cerebellar atrophy of this patient. Ubiquitination is involved in two pathways for the degradation of mutated membrane proteins. During biosynthesis in endoplasmic reticulum, the unfolded protein response transports misfolded protein to the cytoplasm where its ubiquitination leads to the proteasome [36], and if a protein is trafficked properly but unstable or misfolded, its ubiquitination leads to autophagy and the lysosome [37]. The role of ubiquilin-4 is not well-studied, but it was discovered as a result of its binding to ataxin-1, whose poly-glutamine expansion causes spinocerebellar atrophy type1 [38]. Ubiquilin-4 is reported to recruit ubiquilin-1 to the proteasome [39]. The homolog *UBQLN2* (ubiquilin-2) on the X chromosome has several gender-independent dominant mutations in a collagen-like proline-X-X repeat that cause ALS-FTD (amyotrophic lateral sclerosis-frontotemporal dementia) [40]. Just upstream of the proline mutations, in a sequence conserved in ubiquilins, is a homozygous *UBQLN2* missense variant, p.Thr467Ile, found in a female FTD patient [41]. Interestingly, the ubiquilin-2 sequence 461-GLQTLA**T**EAPGLIPS-475 aligns with ubiquilin-4 475-GLQTLQT**E**APGLVPS-489, and therefore the UDP patient’s heterozygous variant and the FTD-associated homozygous variant are adjacent in the aligned sequence. The functional consequences of the *UBQLN4* p.Glu482Lys variant are not known, but substitutions of negatively charged glutamate with positively charged lysine would be expected to be more disruptive than a threonine to isoleucine change, and the sequence is highly conserved. If the aligned mutations both impair their respective proteins, it would be credible for the *UBQLN4* mutation to reduce the capacity of neurons to dispose of ubiquitinated, misfolded Na,K-ATPase, provided that the *ATP1A3* and *UBQLN4* genes are expressed in the same neurons. In the Allen Brain Atlas [42], the mouse cerebellum shows similar cellular RNA distributions of the two gene products: molecular layer neurons and scattered granular layer neurons, Purkinje cells, and neurons of the dentate nucleus, but not cerebellar granule cells. Mouse Purkinje cell expression of *Atp1a3* is particularly high, a condition that could promote accumulation of misfolded mutated Na,K-ATPase and lead to cell death if the *UBQLN4* variant impaired degradation.

### Redundancy in *GPRIN1*

**Fig 4:**
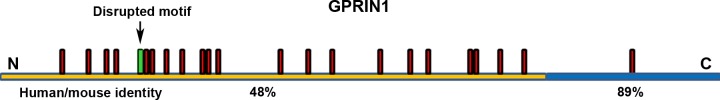
Repeated motifs in GPRIN1. GPRIN1 (formerly known as GRIN1 until the name conflicted with the official gene name of an NMDA glutamate receptor) has sequence characteristics of a relatively unstructured protein, and in human it has 23 copies, of differing fidelity, of a six amino acid motif typified by KEDPGS. The diagram shows the distribution of motifs (red bars) and the location of an abrupt change of the degree of conservation between human and mouse. The less-conserved segment is likely to be relatively unstructured, and the conserved segment is likely to be compactly folded. One motif copy (green bar) is homozygously deleted in the patient.

In the patient one identical allele of *GPRIN1* (G-protein-regulated inducer of neurite outgrowth 1) was inherited from each heterozygous parent. In variant databases, the in-frame deletion is a known variant (rs371149640) in a cluster of similar short deletions and SNPs with no associated pathogenesis. The deletion disrupts a motif that is present in multiple copies (Fig 4). The redundancy of the deleted motif is expected to reduce the likelihood that the deletion is causative of disease. The repeated motifs and other features of the *GPRIN1* protein sequence suggest a scaffolding function consistent with the protein’s role in neurite outgrowth and the association of receptors with membrane [43–45]. Detail is provided in Figure A in S1 File.

### Implications for dystonia circuitry

Adult RDP patients generally manifest fixed dystonia [3], and it would be expected to accompany the other RDP symptoms in this patient. Persistent or recurring dystonia generally develops in AHC patients as well. There is much evidence that dystonia circuits include the cerebellum [46]. Purkinje cell ablation and cerebellectomy block dystonic symptoms in animal models; cerebello-thalamo-cortical tracts are altered in diffusion tensor imaging in patients; in RDP the dentate nucleus showed extensive neuropathology; and Na,K-ATPase inhibition causes abnormal burst firing of Purkinje neurons [17,47–50]. The near-absence of dystonia in the presence of severe cerebellar atrophy aligns with the importance of cerebellar output to dystonia pathophysiology.

## Supporting Information

S1 File Supplementary data. Table A. Psychiatric evaluation. Table B. Neuropsychological evaluation. Figure A. Redundancy of the deleted motif in *GPRIN1*.
(PDF)

S1 Video Neurological exam of the patient. (MP4)
