# Supplementary material for: ATP1A3 Mutation in Adult Rapid-Onset Ataxia
Source: PLoS One. 2016 Mar 18;11(3):e0151429. doi: 10.1371/journal.pone.0151429 (PMC4798776; doi:10.1371/journal.pone.0151429)
Supplement: S1 File — Table A. Psychiatric evaluation. Table B. Neuropsychological evaluation. Figure A. Redundancy of the deleted motif in GPRIN1. (PDF) [file pone.0151429.s001.pdf]

Table A. Psychiatric evaluation

Test	Score
**CICI**	
**DSM-IV**	No diagnostic criteria met
**ICD-10**	No diagnostic criteria met
**Ham-D**	6 (within normal limits)
**Ham-A**	9 (within normal limits)
**Y-BOCS**	0

The psychiatric tests administered were:
Composite International Diagnostic Interview (CIDI-Auto version, previously used in studies of patients with DYT1)[9, 10]
Hamilton Depression Rating Scale (HAM-D)[11]
Hamilton Anxiety Scale (HAM-A)[12]
Yale-Brown Obsessive-Compulsive Scale (Y-BOCS)[13]

Table B. Neuropsychological evaluation

INTELLECTUAL		
**WASI-II**	**Raw**	**SS, T / PR**
**Full Scale IQ Estimate**	--	88 / 21st
**Verbal IQ / VCI Estimate**	--	90 / 25th
**Vocabulary**	36	47 / 37th
**Similarities**	26	40 / 16th
**Performance IQ / PRI Estimate**	--	89 / 23rd
**Block Design**	32	41 / 19th
**Matrix Reasoning**	19	46 / 34th
**Raven's Progressive Matrices**	39	79 / 8th
**LANGUAGE**		
**COWA**	**Raw**	**T / PR**
**Letter Fluency (FAS)**	30	38 / 13th
**Category Fluency (Animals)**	23	49 / 47th
**Boston Naming Test**	55	42 / 21st
**Correct with Phonemic Cues**	57	--
**ATTENTION, SPEED, EXECUTIVE SKILLS**		
**WAIS-III**	**Raw**	**T, ScS / PR**
**Digit Span (overall)**	10	5 / 5th
**Forward Span**	4	30 / 2nd
**Backward Span**	4	44 / 30th
**Digit Symbol Coding**	16	2 / 0.4th
**SDMT**	**Raw**	**Z / PR**
**Written**	12	-5.04 / <0.1th
**Oral**	24	-3.58 / <0.1th

cont.

Trail Making Test	Seconds	T / PR
Trails A	100	2 / <0.1th
Trails B	199	19 / 0.1th
**WCST-64**	**Raw**	**T / PR**
Categories Completed	3	>16th
Trials to first category	13	11th – 16th
Total Errors	16	44 / 27th
# Perseverative Responses	11	40 / 16th
# Perseverative Errors	8	43 / 23rd
# Failures to maintain set	1	--
**VERBAL MEMORY**		
**WRAML2**	**Raw**	**SS, ScS / PR**
Verbal Memory Index	--	105 / 64th
Verbal Recognition Index	--	112 / 79th
Verbal Learning # recalled by trial (raw)	7,11,12,13	
Total Words Recalled	43	11 / 63rd
Delayed Recall	12	11 / 63rd
Recognition	40	≥13 / ≥84th
**WRAML2 Story**	**Raw**	**ScS / PR**
Immediate Free Recall	46	11 / 63rd
Delayed Free Recall	46	11 / 63rd
Delayed Recognition	35	11 / 63rd
**VISUAL MEMORY**		
**WRAML2**	**Raw**	**SS, ScS / PR**
Visual Memory Index	--	91 / 27th
Visual Recognition Index	--	75 / 5th
Design Memory		
Total Details Recalled	30	8 / 25th
Delayed Recognition	23	4 / 2nd
**WRAML2 Picture Memory**	**Raw**	**ScS /PR**
Immediate Recognition	30	9 / 37th
Delayed Recognition	32	8 / 25th

Standard Score (SS): mean = 100, SD = 15. Scaled Score (ScS): mean = 10, SD = 3. T-score: mean = 50, SD = 10. Z-score: mean = 0.0, SD = 1. Percentile mean=50. PR=percentile.

The neuropsychologic tests administered were:
Wechsler Abbreviated Scale of Intelligence-II (WASI-II)[14]
Raven's Progressive Matrices[15]
Wechsler Adult Intelligence Scale-III (WAIS-III, selected subtests)[16]
Controlled Oral Word Association (COWA)[17]
Wide Range Assessment of Memory and Learning 2 (WRAML2)[18]
Trail Making Test (A&B)[19]
Symbol Digit Modalities Test (SDMT)[20]
Wisconsin Card Sort Test -64 (WCST-64)[21]

Figure A. Redundancy of the deleted motif in *GPRIN1*

human (2nd methionine)

mouse

```
Query  42   MRDYCPSQQKASPAPPRHTPDQSPGMESRHRSPSGAGEGASCSDGPRGSLACPSPTCFSP  101
            MRD C S  KA PAPPRH  DQS GM+ RH S SGA EGASCS+ P GSLACPSP C
Sbjct  1    MRDCC-SSPKAIPAPPRHALDQSLGMDPRHTSSSGAAEGASCSERPAGSLACPSPNC---  56

Query  102  QESPSKETLEAHGASISGTPEATTSGKPEPVSSVKTEPKSSDDRNPMFLEKMDFKSSKQA  161
              SP  ET  AHGA  S     T  GKPEP+SS +  P +S+ RNP+F  KMD  S KQA
Sbjct  57   --SPLPETPRAHGALTSDNSGTTLFGKPEPMSSAEATPTASEIRNPVFSGKMDGNSLKQA  114

Query  162  DSTSIGKEDPGSSRKADPMFTGKAEPEILGKGDPVAPGRMDPMTVRKEDLGSLGKVDPLC  221
            DSTS  KE+ GS R  + M  GKAEP I GKG+P   GR+D      E+ GSLGKVD  C
Sbjct  115  DSTSTRKEEAGSLRNEESMLKGKAEPMIYGKGEPGTVGRVDCTASGAENSGSLGKVDMPC  174

Query  222  SSKTYTVSPRKEDPGSLRKVDPVSSDKVDPVFPRKEEPRYSGKEHPVSSEKVAPTSAEKV  281
            SSK   VSP  ++ GSLRKV+ +SS K+D   P+ E   +S +E P S
Sbjct  175  SSKVDIVSPGGDNAGSLRKVETISSGKMD---PKTENVMHSRRERPGS------------  219

Query  282  DLVLSGKRDPGPSGKADPMPLESMDSASTGKTEPGLLGKLIPGSSGKNGPVSSGTGAPGS  341
                +G+ D     + D  P ++ DSAST KT+P   GKL PGSSGK   VSS T AP +
Sbjct  220  ----TGEGDLVSLRENDMKPPDNTDSASTKKTDPEFSGKLTPGSSGKTELVSSVTVAPVT  275

Query  342  LGRLDPTCLGMADPASVGNVETVPATKEDSRFLGKMDPASSGEGRPVSGHTDTTAS----  397
               ++P C G A PA+VGN ET+ + K+D + LGK +  SSGEG  VS     T S
Sbjct  276  SENVNPVCSGGAGPAAVGNSETLSSVKKDPQLLGKKEAVSSGEGGSVSVRMAETVSARQP  335
```

deleted segment

48% identity, many

motifs

```
Query  398  -----AKTDLTSLKNVDPMSSGKVDPVSLGKMDPMCSGKPELLSPGQAERVSVGKAGTVS  452
                 AKTD TS  +  P  SG+ DPVSL   + +   KPE LS GQAERVS+ K  T+S
Sbjct  336  EGMFPAKTDSTSSNSTGP--SGRADPVSLRNSELVSPVKPERLSSGQAERVSLVKTETLS  393

Query  453  PGKEDPVSSRREDPISAGSRKTSSEKVNPESSGKTNPVSSGPGDPRSLGTAGPPSAVKAE  512
             GKEDP SSRR D  +      +S+K NPESSGKT+  SS  GD RSLGT G  SA KAE
Sbjct  394  SGKEDPRSSRRVDHTTVTGNMQTSQKGNPESSGKTDLGSSSSGDTRSLGTWGSLSAAKAE  453

Query  513  PATGGKGDPLSSEKAGLVASGKAAPTASGKAEPLAVGKEDPVSKGKADAGPSGQGDSVSI  572
              T GKGDP   +KA L AS K  P AS KA   + GK + VS G+ DA        +++
Sbjct  454  -VTEGKGDPQPWKKASLPASEKTDPLASSKAGSASQGKAETVSPGEVDA--------MTL  504

Query  573  GKVV-STPGKTVPVPSGKVDPVSLGKAEAIPEGKVGSLPLEKGSPVTTTKADPRASGKAQ  631
            GK V ++ GKT  V  GKVD ++  +AE IPE +      EKG+PV +T+ D  A+G  +
Sbjct  505  GKTVPTSSGKTALVSPGKVDLMTSERAEGIPELQAS----EKGNPVNSTRVDTGATGSTE  560
```

```
Query  632   PQSGGKAETKLPGQEGAAAPGEAGAVCLKKETPQASEKVDPGSCRKAEPLASGKGEPVSL  691
             P+SG K  T++P   GA +PG+     L+KE PQ SEK DP   RK +P  +   EPVSL
Sbjct  561   PKSGVKVITQIP---GATSPGKVETPSLQKEQPQLSEKTDP--SRKVDPPTT--VEPVSL  613

Query  692   GKADSA-PS-RKTESPSLGKVVPLSLEKTKPSSSSRQLDRKALGSARSPEGARGSEGRVE  749
             GKADSA PS RK ES +  K VP + +K   +SS RQ D     SA+ P+    S G +
Sbjct  614   GKADSASPSPRKAESQTSAKTVPQAPDKA--TSSLRQSDGTPYSSAQ-PQRDTRSIGSL-  669

Query  750   PKGEPVSSTEASSLGQKDLEAAGAERSPCPEAAAPPPGPRTRDNFTKAPSWEASAPPPPR  809
             P+ EP +ST      QKDL AA A++SP  EAAAPPPGPRTRDNFTKAPSW+A A PPPR
Sbjct  670   PEREPSASTS-----QKDLAAAAAQKSPSAEAAAPPPGPRTRDNFTKAPSWDAGA-PPPR  723

Query  810   EDAGTQAGAQACVSVAVSPMSPQDGAGGSAFSFQ----AAPRAPSPPSRRDAGLQVSLGA  865
             EDAGTQAGAQACVSVAVSPMSPQDGAGG AFSFQ    A   AP PPSRRDAGLQVSLGA
Sbjct  724   EDAGTQAGAQACVSVAVSPMSPQDGAGGPAFSFQAAPRAPSPAPRPPSRRDAGLQVSLGA  783

Query  866   AETRSVATGPMTPQ----AAAPPAFPEVRVRPGSALAAAVAPPEPAEPVRDVSWDEKGMT  921
             AETRSVATGPMTPQ     A PP FPEVRVRPGS LAAA+AP E  EPVRDVSWDEKGMT
Sbjct  784   AETRSVATGPMTPQAAAPPAVPPVFPEVRVRPGSVLAAALAPQEATEPVRDVSWDEKGMT  843

Query  922   WEVYGAAMEVEVLGMAIQKHLERQIEEHGRQG--APAPPPAARAGPGRSGSVRTAPPDGA  979
             WEVYGA+MEVEVLGMAIQKHLERQIEEHGRQG  APAPPPA RAGPGR+GSVRTAP +GA
Sbjct  844   WEVYGASMEVEVLGMAIQKHLERQIEEHGRQGAPAPAPPPAVRAGPGRAGSVRTAPAEGA  903

Query  980   AKRPPGLFRALLQSVRRPRCCSRAGPTAE  1008
             AKRPPGLFRALLQSVRRPRCCSRAGPTAE
Sbjct  904   AKRPPGLFRALLQSVRRPRCCSRAGPTAE  932
```

89% identity, one motif

Human and mouse GPRIN1 proteins were aligned with blastp (NCBI). Identical and homologous residues are shown between the lines. Homology is bimodal: the first ¾ of the sequence has lower homology, while the last ¼ has higher homology but still has four indels between human and mouse. The low inter-species homology of the first ¾ of the protein reflects a lack of selective pressure. This is likely to indicate intrinsic disorder, a characteristic of some protein-binding domains. GPRIN1 protein is also proline-rich (14%), typical of proteins with extended or unstructured domains.

The in-frame deletion (rs371149640) inherited from both heterozygous parents was in a cluster of very similar short deletions (rs142779818, rs550332435, rs386695335, including one that is very frequent (28%); others are reported in Ensembl). At the same locus is a cluster of SNPs (6 with incidence >1% and 3 with incidence < 1%) with no associated pathogenesis. The underlying cause of the cluster of deletion variants is likely to be an adjacent 18 nucleotide base repetition at that position (18 out of the 24 deleted bases) (not shown).

Repeated motifs (red) of unknown significance are found predominantly in the less conserved part of the protein. There are as many as 23 repetitions in human that vary in fidelity, but the majority can be represented as [K/R][E/ϕ] [D/E/X]P[G/V]S, where ϕ is any hydrophobic amino acid. The patient deletion, which eliminates one of two adjacent motifs, is shown in green.
